# Quantitative Fitness Analysis Identifies *exo1∆* and Other Suppressors or Enhancers of Telomere Defects in *Schizosaccharomyces pombe*

**DOI:** 10.1371/journal.pone.0132240

**Published:** 2015-07-13

**Authors:** Siddharth Narayanan, Marion Dubarry, Conor Lawless, A. Peter Banks, Darren J. Wilkinson, Simon K. Whitehall, David Lydall

**Affiliations:** 1 Institute for Cell & Molecular Biosciences, Newcastle University Medical School, Newcastle upon Tyne, NE2 4HH, United Kingdom; 2 High Throughput Screening Facility, Newcastle Biomedicine, Newcastle University, Newcastle upon Tyne, NE2 4HH, United Kingdom; 3 School of Mathematics & Statistics, Newcastle University, Newcastle upon Tyne, NE1 7RU, United Kingdom; Tulane University Health Sciences Center, UNITED STATES

## Abstract

Synthetic genetic array (SGA) has been successfully used to identify genetic interactions in *S*. *cerevisiae* and *S*. *pombe*. In *S*. *pombe*, SGA methods use either cycloheximide (*C*) or heat shock (*HS*) to select double mutants before measuring colony size as a surrogate for fitness. Quantitative Fitness Analysis (QFA) is a different method for determining fitness of microbial strains. In QFA, liquid cultures are spotted onto solid agar and growth curves determined for each spot by photography and model fitting. Here, we compared the two *S*. *pombe* SGA methods and found that the *HS* method was more reproducible for us. We also developed a QFA procedure for *S*. *pombe*. We used QFA to identify genetic interactions affecting two temperature sensitive, telomere associated query mutations (*taz1Δ* and *pot1-1*). We identify *exo1∆* and other gene deletions as suppressors or enhancers of *S*. *pombe* telomere defects. Our study identifies known and novel gene deletions affecting the fitness of strains with telomere defects. The interactions we identify may be relevant in human cells.

## Introduction

Genetic interactions (GIs) arise when the function of one gene is affected by the function of another [1]. In budding yeast, synthetic genetic array (SGA) methodology has been used to characterise GIs on a genome-wide scale [2–4]. SGA uses large-scale robotic procedures for mating and sporulation carried out on solid agar media to generate double mutant colonies and to measure their size [5]. By comparing the size of double mutants it is possible to classify GIs as negative (where double mutant colonies are smaller than expected), positive (larger than expected) or neutral [6–10]. Genome-wide SGA screens performed using the budding yeast *S*. *cerevisiae* have categorised gene subsets based on functionality [5, 11–13]. Techniques similar to budding yeast SGA have been developed for *E coli* [14, 15] and *S*. *pombe* [16, 17].

Quantitative fitness analysis (QFA) is another high throughput method for measuring fitness phenotypes in budding yeast. In QFA, strains are cultured in liquid media, spotted onto solid agar plates and growth is monitored by time course photography. A logistic growth curve model is fitted to the data to infer fitness phenotypes such as maximum growth rate or maximum doubling potential [18–20]. QFA has been successfully used to establish GIs affecting telomere related query mutations [19].

Hundreds of mutations interacting both positively and negatively with mutations affecting telomere-binding proteins such as Cdc13 in budding yeast have been identified using QFA [19]. We were therefore interested to try to apply QFA to *S*. *pombe* telomere-defective strains to permit us to compare and contrast the genetic interactions we see in the two yeast species. Fission yeast is evolutionarily distant from budding yeast and the comparisons have the potential to identify interactions conserved in metazoans [21–23]. Telomere structure is similar in budding and fission yeast and key proteins associated with telomeres in these yeasts are functionally conserved in mammals [21, 24, 25]. For example, the single strand DNA-capping protein Pot1 in human and fission yeast contains OB-folds as do budding yeast Cdc13 and human CTC1. Furthermore fission yeast *pot1-1* mutants confer similar phenotypes to budding yeast *cdc13-1* mutants [26]**.** Telomere defects in human cells are relevant to ageing and carcinogenesis, for example telomere degradation and fusion events occur during carcinogenesis and ageing [27–32].

There are two published methods to generate *S*. *pomb*e double mutant strains during SGA, using either heat shock (*HS*) at 42°C or cycloheximide (*C*) as a critical selective step [16, 17]. Cyclohexamide-based SGAs require the genetically engineered ‘pombe epistatic marker 2’ (PEM2) parental strain and, in this background, growth in presence of cyclohexamide serves as both anti-diploid and mating-type selection [16, 33–36]. The *HS* based method does not require a specific genetic background [17] but uses high temperature to kill vegetative cells and therefore select for spores. In this paper we set out to test the different methods for carrying out genome-wide SGA screens in *S*. *pombe* and to develop a QFA procedure for *S*. *pombe*. We used QFA to identify known and novel suppressors and enhancers of *S*. *pombe* telomere defects.

## Results

### Comparing SGA Methods

To evaluate the two different *S*. *pombe* SGA methods and to choose the best ‘neutral’ mutation, a gene deletion library (2936 *S*. *pombe yfgΔ*, **y**our **f**avourite **g**ene deletions, version 3) [37] was crossed with query mutations using two SGA methodologies [3]. In principle, each of the two *S*. *pombe* SGA methods to generate double mutants should result in a similar pattern of GIs. To test if this is the case we first carried out SGAs with *his3Δ*, *ura5Δ and his7Δ* as comparatively ‘neutral’ query mutations using heat shock (*HS*) [17] or cyclohexamide (*C*) based methods [16, 38].

**Fig 1:**
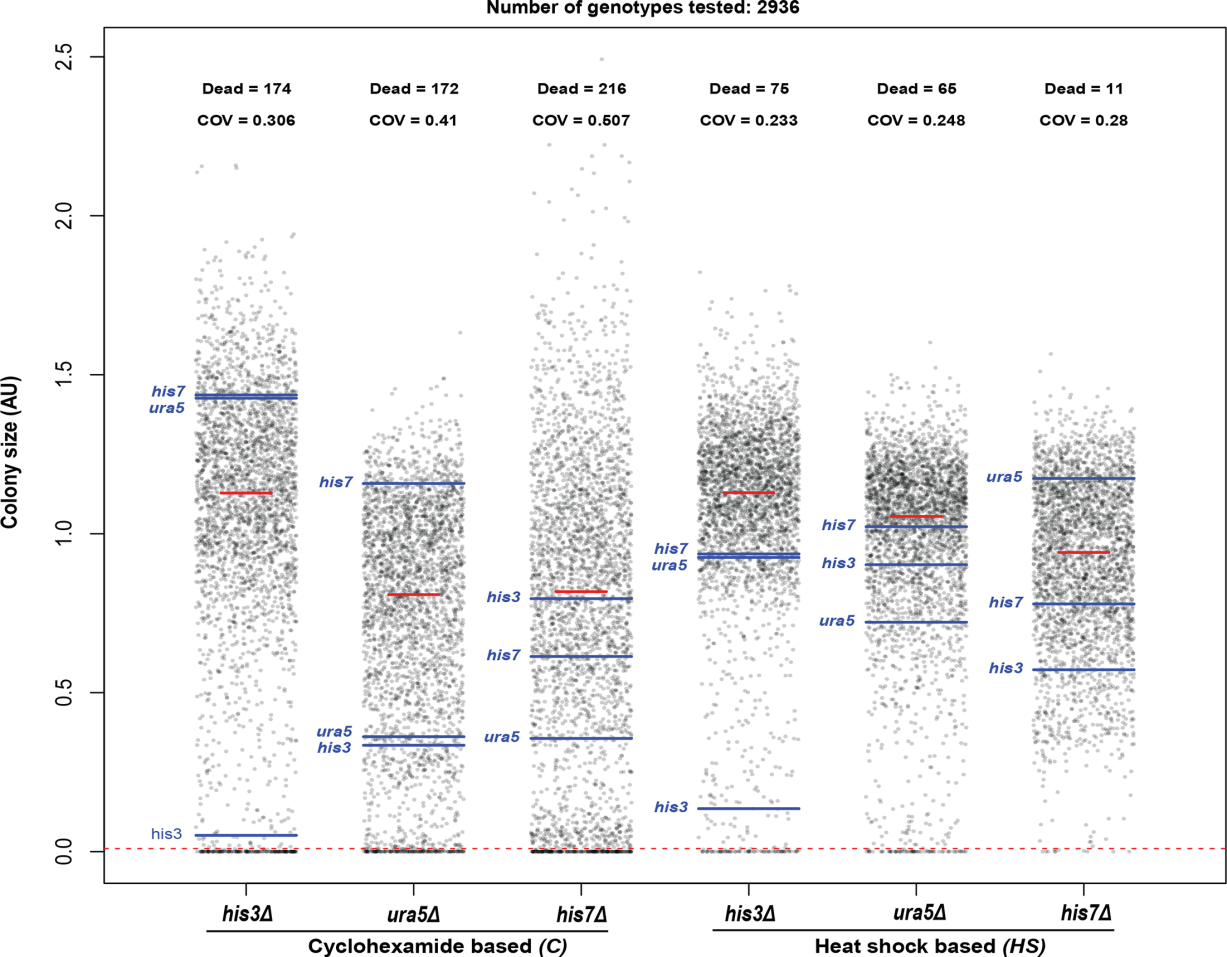
Comparison of *HS* and *C* based SGA methods. *his3Δ*, *ura5Δ* and *his7Δ* mutations were crossed with a deletion library either using *C* or *HS* method, and colony size measured. Strip charts comparing colony size distributions of double mutants on SGA final selection plates are shown. The horizontal blue lines represent library deletion strains and the red line is the median value of each experiment. Colony sizes were scaled relative to the median of the overall distribution across all six screens. The horizontal dotted red line is the threshold below which colonies were classified as "dead", and is based on a colony size less than 1% of the median across all 6 SGAs. The coefficient of variation (COV) for colony sizes above the cut-off is shown.

Fig 1 is a summary of the fitnesses we observed in six independent SGA experiments. Unexpectedly, we found that the fitness rankings for *his3Δ*, *ura5Δ and his7Δ* SGAs were different to each other in the rich media we use for SGA. Using either *HS* or *C* methods we observed that, on average, the *his3∆* strains were fitter than *ura5∆* or *his7∆* strains. We also observed that overall colony size distribution was different between *HS* and *C* methods (Fig 1). Overall, colony size distribution in *his3Δ*, *ura5Δ and his7Δ* SGAs appeared tighter using the *HS* method compared to the *C* method (Fig 1). This tighter spread was reflected by lower coefficient of variation (COV) values for the *his3Δ*, *ura5Δ* and *his7Δ* SGAs with the *HS* method versus the *C* method. We also observed that the proportion of very low fitness, or "dead" double mutant strains was lower for the *HS* method in all cases (Fig 1).

Finally, we looked for evidence of genetic linkage to *his3Δ*, *ura5Δ* or *his7Δ* to determine SGA success. If a haploid *his3*::KANMX strain is crossed with a *his3*::NATMX strain, haploid double deletion progeny should not arise after mating and sporulation. However we should be able to combine the *his3∆* allele with all other unlinked gene deletions. The position of the *his3Δ* strains, as indicated by the blue line, is close to zero in both *his3Δ* SGA methods suggesting that these SGAs were most successful (Fig 1). Importantly, the *his3Δ* parental strain generated *his3Δ ura5Δ* double mutants and *his3Δ his7Δ* double mutants. The *ura5∆* and *his7∆* strains showed less strong evidence for genetic linkage (and by implication SGA success). We conclude that *his3∆* is a better choice than *his7∆* or *ura5∆* to use as a neutral mutation for control SGAs. Overall, based on these six SGAs we also conclude that the *HS* method generates more viable double mutants than the *C* method, and that the double mutants within the *HS* method are more similar in fitness to each other (Fig 1).

**Fig 2:**
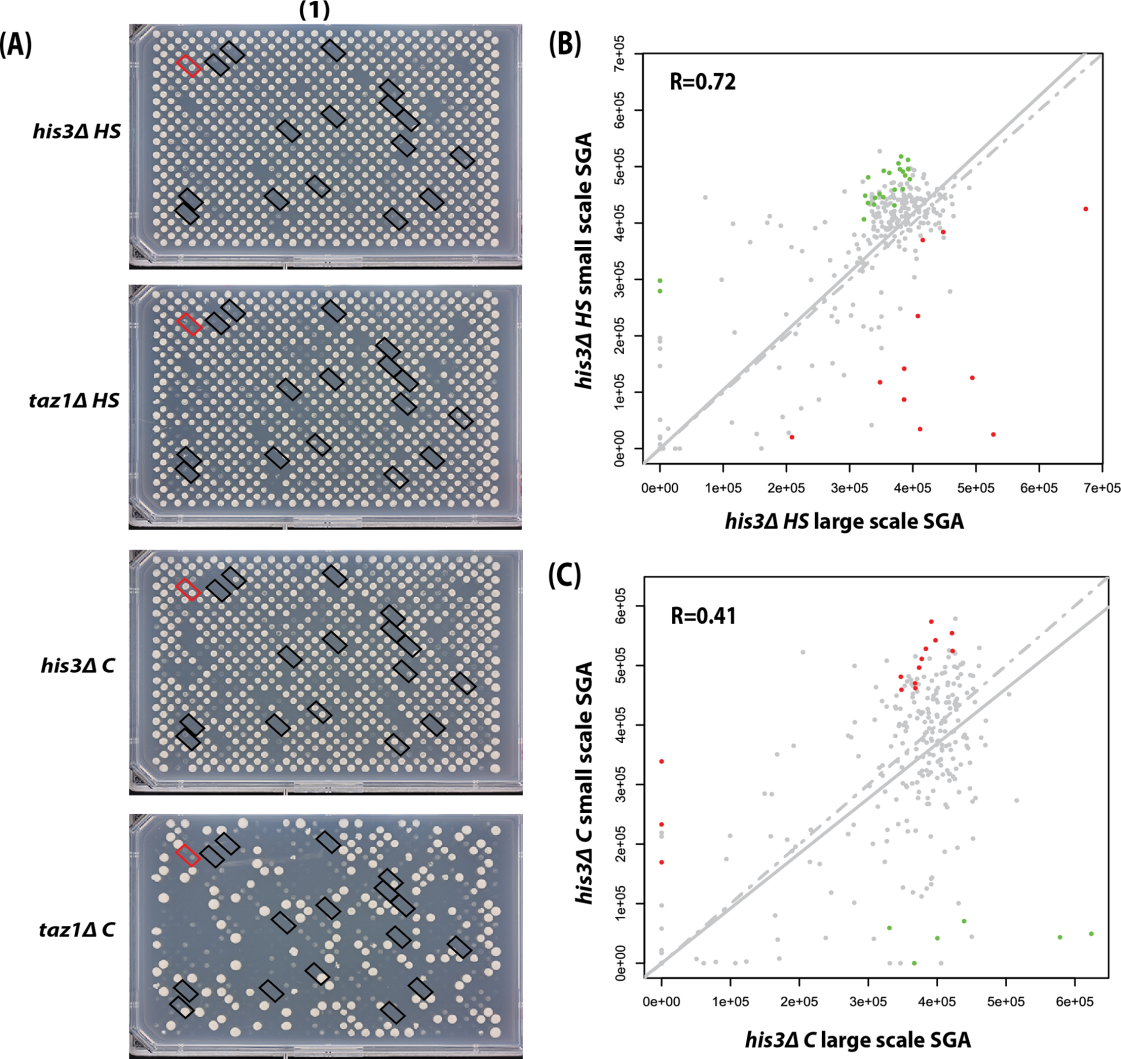
Small scale SGA demonstrates the *taz1Δ* query mutation survives the *HS* method better than the *C* method. *his3Δ* and *taz1Δ* strains were crossed with a subset (n = 308, Worksheet B in S1 Tables; many telomere-related) of gene deletions from the deletion library using the two methods. **(A)** Images of final selection plates from the *HS* and *C* methods are shown. The double mutants were grown at 30°C after 3 days. One plate (of a total of 4) having two independent replicates was processed for each method. The 16 diagonal black rectangular boxes indicate a proportion of double deletion pairs showing growth defects in the four SGA plates. The red rectangular box highlights the *sde2Δ* location. **(B)** Correlation scatter plots comparing the *his3Δ* small scale SGA and the *his3Δ* large scale SGA using the *HS* method for 308 genes **(C)** Same as (B) but comparison made using the *C* method.

We next performed a small scale SGA on a telomere-defective *taz1Δ* query strain and a neutral *his3Δ* control strain. For this experiment a small library of deletion mutations (n = 308, Worksheet B in S1 Tables), many of which were shown previously to interact with *taz1Δ* mutation, was used. At permissive temperatures (30°C) we generated 8 independent replicates of each genotype arranged on four separate plates. Fig 2A shows images of four SGA final selection media plates. Double mutants were arrayed in a 768 colony format with 308 deletion mutations arranged in pairs and surrounded by a neutral mutation (*mug134Δ*) in pairs. A true synthetic lethal interaction is inferred when each of a pair of replicate double deletion mutants does not form colonies. SGA artefacts can be inferred if colony pairs carrying the same mutations do not behave similarly on the same plate (viable or non-viable) or across plates (S1 Fig).

In the *his3Δ HS* SGA experiment, absence of particular *his3Δ* progeny pairs was largely concordant within each plate (Fig 2A, top row- the black diagonal boxes highlight all consistently ‘synthetic lethal’ interactions) or within the eight repeats across all four *his3Δ HS* SGA plates (S2 Fig, top row). The *taz1Δ HS* experiment generated a broadly similar synthetic lethal growth pattern (Fig 2A, second row) and was largely consistent when compared with the *his3Δ HS* SGA (correlation, R = 0.59; Panel B in S3 Fig). It has been reported that *sde2∆* is synthetically lethal with *taz1∆* [39] and reassuringly we confirmed this result (highlighted in red, Fig 2A, top 1 panels). We concluded that the *HS* based SGA method is reproducible and could identify meaningful genetic interactions.

We next conducted a *taz1Δ* SGA using the *C* method. We found that the *his3Δ C* experiment resulted in a higher proportion of inviability and inconsistency across the eight replicates in comparison with the *HS* method (Fig 2A, third panel and S2 Fig). Despite observing the synthetic lethality with *sde2∆*, the *taz1Δ C* method generated poorly growing or dead strains extremely frequently (Fig 2A, bottom panel).

Finally, the *his3Δ* small scale (Fig 2A) and large scale SGAs (Fig 1) were compared to test reproducibility across experiments performed at different times (Fig 2B and 2C). Replicates of screens carried out using the *HS* methods were consistent, with a strong correlation between colony sizes (R = 0.72) whereas a more moderate correlation (R = 0.41) was observed with the *C* method (Fig 2B and 2C). Overall, we found the *HS* method more reproducible and used it for standardising QFA in *S*. *pombe*.

### QFA Identifies Known and Novel GIs among Telomere Defective Mutants

The SGA technique uses colony size to determine strain fitness [2, 11, 40]. Quantitative Fitness Analysis (QFA) is different and measures fitnesses by analysing growth curves [19, 20]. QFA growth curves are very similar to those observed in liquid culture, with clear exponential and saturation phases [18]. Using QFA many more cultures can be examined in parallel than is practical using liquid cultures. Furthermore, spotted QFA provides more accurate fitness measurements than can be measured using pinned cultures [19]. Therefore we wanted to assess QFA as a means of identifying and quantifying GIs for *taz1Δ* and *pot1-1* telomere capping mutations in *S*. *pombe*.

QFA was first performed on double mutants obtained after crossing *his3Δ* and *taz1Δ* mutations to a small deletion library by SGA (n = 308, Worksheet B in S1 Tables). The *taz1Δ* strain is cold sensitive at 20°C [41, 42]. Therefore we first cultured double mutants in liquid under permissive conditions (30°C) before measuring fitness by QFA under restrictive conditions (20°C).

**Fig 3:**
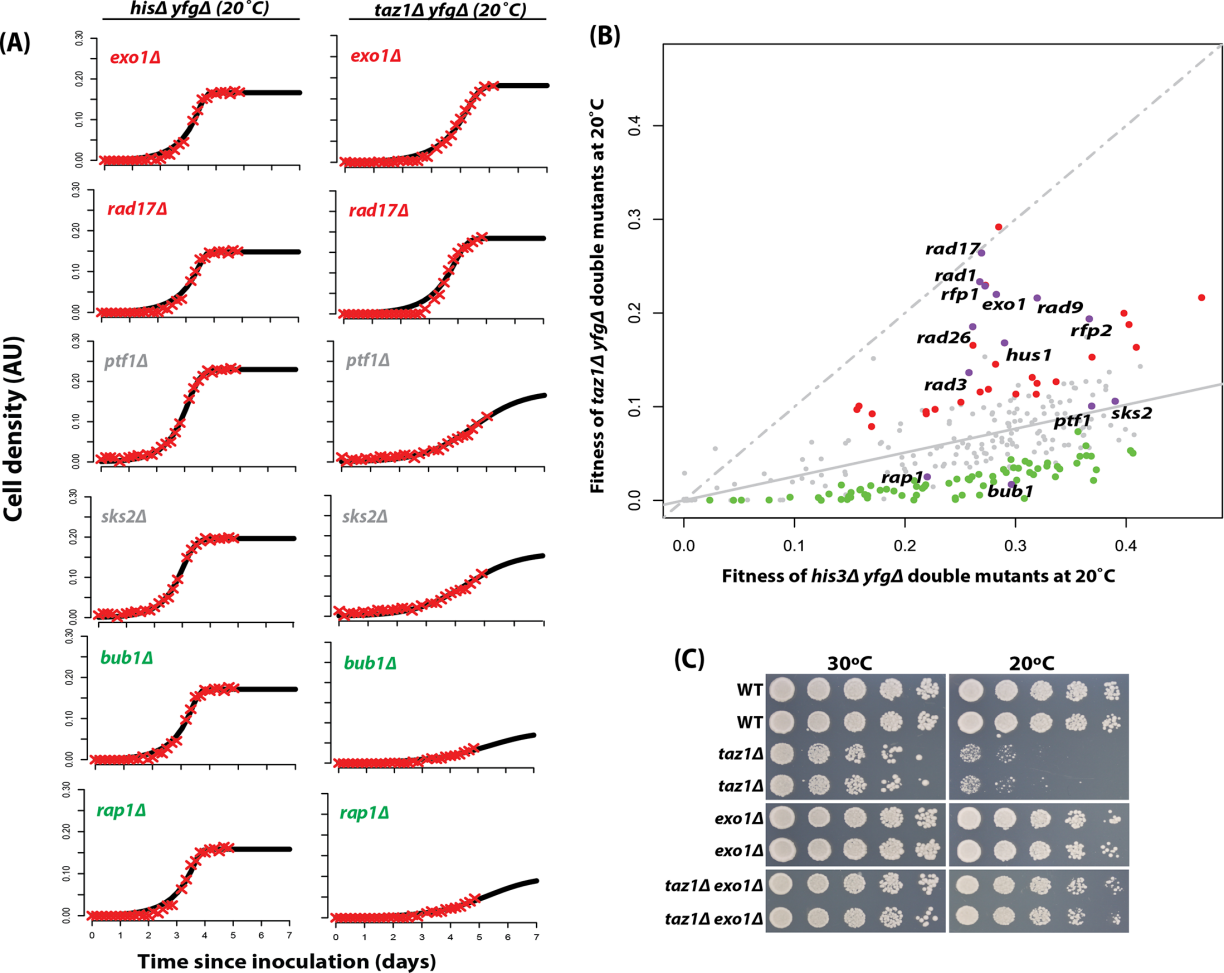
Small scale QFA identifies previously known telomere associated genes as suppressors of *taz1Δ* cold sensitivity. (A) QFA growth curve comparisons of representative *his3Δ yfgΔ* and *taz1Δ yfgΔ* strains at 20°C for 5 days. (B) Four replicates of double mutants from the *his3Δ* and *taz1Δ* small scale SGA using the *HS* method were first cultured in liquid at 30°C and then spotted. Growth of *taz1Δ yfgΔ* and the *his3Δ yfgΔ* double mutants was measured at 20°C. Numerical area under the curve (nAUC) values were used as measures of double mutant fitness. Gene deletions that significantly enhance (green) or suppress (red) the *taz1Δ* defect, in comparison with the *his3Δ* mutation are indicated. The line of equal growth (dashed grey) and a population model of expected fitness (solid grey) are indicated. (*C*) The *exo1Δ* (independent of the Bioneer library) was combined with a *taz1Δ* mutation and assessed for growth by manual spot test. The strains were cultured to saturation in 2ml YE5S media at 30°C, a five-fold serial dilution generated and spotted on YE5S plates. Strains were incubated in the same incubator at the same time and at indicated temperatures for 3–6 days before being photographed.

In Fig 3A, we show growth curves for some representative *his3Δ yfgΔ* and *taz1Δ yfgΔ* double mutant strains, some of which were previously known to interact with *taz1* ^*+*^. The *taz1Δ exo1Δ* and *taz1Δ rad17Δ* strains grew as well as the equivalent *his3Δ* strains at 20°C whereas *taz1Δ bub1Δ* and *taz1Δ rap1Δ* strains grew relatively poorly. The *taz1Δ ptf1Δ* and *taz1Δ sks2Δ* had intermediate fitnesses. Fig 3B shows fitnesses of all *his3Δ* and *taz1Δ* strains as a scatter plot. A comparison of the *his3Δ yfgΔ* and *taz1Δ yfgΔ* strain fitnesses at the permissive temperature (30°C) showed that *taz1Δ* mutants were as fit as the *his3Δ* mutants (the solid grey line superimposing over the line of equal fitness (S4 Fig)). At 20°C, as expected, *taz1Δ* mutants exhibited growth defects relative to *his3Δ* mutants [43]. Interestingly, a number of *taz1Δ yfgΔ* strains grew significantly better than expected, given the fitness of the equivalent *his3Δ yfgΔ* mutation at 20°C (red points, Fig 3B). These *yfgΔ* gene deletions can be classified as *taz1Δ* suppressors. There were also a proportion of *taz1Δ yfgΔ* strains that grew worse than expected and these can be classified as *taz1Δ* enhancers (green points, Fig 3B). The *taz1Δ yfgΔ* strains close to the regression line (neither suppressors nor enhancers) showed no evidence of genetic interaction. The fitness plot in Fig 3B highlights the positions of known suppressors of *taz1Δ* cold sensitivity, such as members of the 9-1-1 complex (*rad9Δ*, *rad1Δ* and *hus1Δ)*, the clamp loader (*rad17Δ)*; and gene deletions of the ATR kinase *RAD3* and its interacting partner *RAD26* [44]. Other known interactions with *taz1* ^*+*^ (enhancers *bub1Δ* and *rap1Δ*) [41, 45] were also identified. Interestingly, deletion of the exonuclease gene *EXO1* was identified as one of the significant suppressors of *taz1Δ* cold sensitivity (Fig 3B). Importantly, we confirmed this novel observation by spot tests of strains generated by tetrad dissection (Fig 3C). Therefore we conclude that QFA is useful for identifying suppressors and enhancers of the *taz1∆* cold sensitive phenotype.

To gain further insights into the *S*. *pombe* telomere cap, we performed a large scale QFA on the temperature sensitive mutant *pot1-1* (protection of telomere) [26]. Double mutants were cultured at 30°C for two days before measuring their fitness at 37°C. This temperature (37^°^C) was chosen because the fitness of *pot1-1* mutants is about half of wild type (S5 Fig). We also assessed the value of a pinned QFA experiment, to determine if we could observe temperature dependent fitness defects of the *pot1-1* strains after pinning, but we observed no strong differences between *pot1-1* and wild type strains between 38°C and 41°C (S6 Fig). This suggests that spotted culture QFA is necessary to observe the temperature sensitive fitness defect in *pot1-1* strains. We did observe that all *POT1* and *pot1-1* strains decreased in fitness as temperatures increased (S6 Fig).

**Fig 4:**
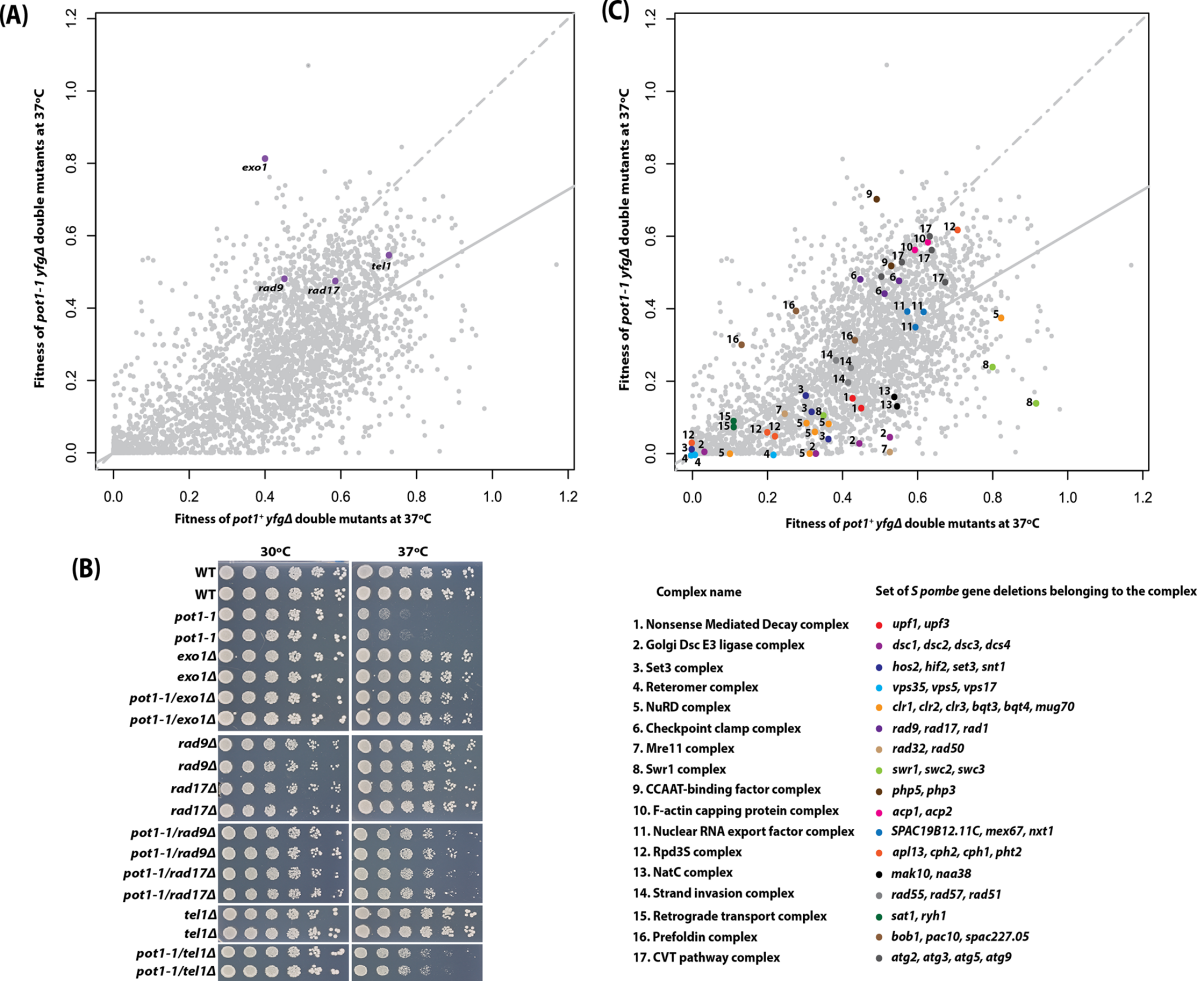
QFA identifies *exo1Δ* and other gene deletions as suppressors or enhancers of *pot1-1* temperature sensitivity. (A) The *S*. *pombe* genome deletion library collection was crossed to the *pot1-1* mutation, or a *POT1* control mutation. Four replicate crosses were carried out for each and the double mutants were first cultured in liquid medium at 30°C and then spotted. The fitness (numerical area under the curve values) of all double mutant cultures measured at 37°C. The line of equal growth (dashed grey) and a population model of expected fitness (solid grey) are indicated. (B) Validation of the *pot1-1* QFA by independent spot tests as described in Fig 3C. (C) Genes grouped based on the hierarchical clustering of GI profiles [10], each number represents a specific complex and the corresponding colour code is a set of genes within that complex.

As expected at 37°C, the *pot1-1* mutation caused a growth defect relative to *pot1* ^*+*^ strains (Fig 4A). We identified *exo1Δ* as one of the strongest suppressors of *pot1-1* temperature sensitivity (Fig 4A). An analogous result is observed in budding yeast where *exo1Δ* is a strong suppressor of *cdc13-1* temperature sensitivity [19, 46]. In order to confirm the results from the *pot1-1* QFA screen, spot tests were performed using manually derived double mutants, where the parental single deletions were constructed independently from those within the deletion library. We confirmed that *exo1Δ* suppresses *pot1-1* temperature sensitivity as do the deletion of genes within the 9-1-1 complex (Fig 4B). Other telomere associated gene deletions (*rad17Δ*, *tel1Δ)* were also identified as suppressors of the *pot1-1* query mutation. However, the QFA data was comparatively noisy in this *pot1-1* experiment compared with the *taz1∆* experiment. For example, there was a wide range of fitness values observed even in the control *pot1* ^*+*^ strains (the x axis). One hypothesis to explain the noisy data is that there is poor growth for many *S pombe* mutant strains at 37^°^C. To test if this might be the case, we chose to highlight the positions of different gene deletions that each affected the same functionally related complexes. Importantly, we found that many gene deletions affecting the same functional complexes [10] clustered together in the data plot (Fig 4C). For example, gene deletions affecting retrograde transport (Complex 15, Fig 4C) grew poorly at 37^°^C irrespective of *pot1-1/ pot1* ^*+*^ status. Overall, the clustering of gene deletions affecting similar processes in Fig 4C suggest that the data are representative of the true effects of each gene deletion on the growth of both *pot1* ^*+*^ and *pot1-1* strains at 37^°^C.

## Discussion

GIs have been measured in different genetic model systems to help understand how cells and organisms function [10, 47–54]. GIs in *S*. *cerevisiae* have been extensively studied [3, 11, 13, 19, 55, 56]. The development of SGA in *S*. *pombe* allows productive comparisons between the genetic networks in budding and fission yeasts [9]. There are two published methods in *S*. *pombe*. The more widely used *C* based method did not work well for us, for reasons that are not clear, while the *HS* method was more reproducible.

QFA measures fitness by analysing growth curves potentially allowing the measurement of more subtle genetic interactions than can be detected by SGA [19, 20]. QFA has been developed in budding yeast to identify subtle suppressor or enhancer GIs in the context of telomere defects. We have established an *S*. *pombe* QFA protocol based on budding yeast protocols. Interestingly, the two yeasts respond differently to the SGA/QFA protocols. For example *S*. *pombe* cell fitnesses are reduced more strongly by storage on agar plates at 4^°^C, time spent in stationary phase (in 96 well liquid cultures) and antibiotic treatments. Therefore we think further optimisation of the QFA protocols are likely necessary. However, even at this stage, QFA can be used to compare genome- wide genetic interactions in fission and budding yeast and this has the potential to identify conserved genetic interactions that are important across all eukaryotic organisms.

Using QFA in *S*. *pombe* we confirmed many known GIs for the *taz1Δ* query mutation such as with gene deletions affecting the 9-1-1 complex [44]. Other novel interactions such as with *exo1Δ* were also identified (Fig 3). Recent studies have demonstrated the role of sumoylation in telomere length regulation [57, 58]. Interestingly, we also identified SUMO-targeted ubiquitin-protein ligases *rfp1* ^*+*^ and *rfp2* ^*+*^ as suppressors of *taz1Δ* cold sensitivity. The human orthologue *RNF4* has been shown to play critical roles in DNA damage response and genome stability [59–62]. Elucidating the roles of *exo1* ^*+*^, *rfp1* ^*+*^ and *rfp2* ^*+*^ at fission yeast telomeres will require further experiments.

Interestingly our *S*. *pombe* QFA identified *exo1∆* as a suppressor of both *taz1Δ* and *pot1-1* induced telomere defects. In budding yeast *exo1∆* suppresses telomere defects because Exo1 is involved in generating ssDNA at uncapped telomeres [19, 46, 63]. The suppression of *taz1Δ* growth defects by *exo1∆* is seemingly at odds with previous findings which indicate that Dna2, not Exo1 is not involved in resecting telomere ends in *taz1Δ* cells [64]. However, in budding yeast there is complex interplay between Exo1 and Dna2 and other factors to control resection at uncapped telomeres [63]. Further experiments will be necessary to clarify the roles of Exo1, Dna2 and other factors at uncapped telomeres in *S*. *pombe*.

Our *S*. *pombe* experiments also showed that deletions affecting the 9-1-1 complex suppressed *pot1-1* temperature sensitivity and these observations correlate with analysis of budding yeast *cdc13-1* mutants [19]. Interestingly, gene deletions affecting the NMD pathway suppress *cdc13-1* [19] but enhance *pot1-1* temperature sensitivity (Fig 4).

*POT1* has been recently identified as a major susceptibility gene for familial melanoma, and is somatically inactivated in chronic lymphocytic leukemia [65–67]. The *S*. *pombe pot1-1* QFA identifies numerous gene deletions which may help in generating exciting hypotheses about roles of individual genes in cells with defective telomeres and potentially therapeutic targets.

## Materials and Methods

### Yeast Strains

All the strains used in this study are listed in Worksheet A in S1 Tables. The strains were generated and grown using standard protocols [68–70]. For the *pot1-1* thermosensitive strain, a marker switch of the KanMX6 module to the HphMX6 module was achieved by transforming the G418^R^ strains with an HphMX6 cassette amplified from pFA6a-HphMX6 plasmid [71]. Positive clones were selected based on growth on YE5S with Hygromycin and inability to grow on YE5S with G418; and tested for thermosensitivity [26].

### SGA Methods

The *HS* and *C* SGA methods were performed as previously described [16, 17]. The Bioneer deletion library collection was used for SGAs (http://us.bioneer.com/products/spombe/spombeoverview.aspx). The deletion library (768 format, 384x2) was grown on YE5S (Yeast extract, 5 supplements) rectangular agar plates with G418. For *S*. *pombe* SGA, a 768 pinning format was used. The cells arrayed on rectangular plates are read from left to right to identify genotypes (column 1, row1 is the top most left corner), two nicks within the plate are towards the top and bottom left corners (S1 Fig). The YE5S agar plates used in SGA had the following drug concentrations; G418 (Geneticin-100 μg/ml), clonNAT (Nourseothricin-100 μg/ml), Hyg (Hygromycin-300 μg/ml, CycH (Cycloheximide-100 μg/ml). EMM_1/2_G (Edinburgh Minimal Media, 0.5 Glutamate; ForMedium, PMD1210) media was used for sporulation.

### S. pombe QFA

The *S*. *pombe* QFA was developed from *S*. *cerevisiae* QFA as previously described [19, 20]. Drug concentrations in media varied depending on whether cells were grown on solid or in liquid media. We observed that 96 well liquid cultures with usual drug concentrations (100 μg/ml G418; 300 μg/ml Hygromycin; 100 μg/ml Nat) grew poorly. Therefore double mutants were cultured in 96-well plates with each well containing 200μl YE5S liquid media with 6.25 μg/ml each (for G418, clonNAT), and 18.75 μg/ml (for Hyg) as final drug concentration were used (G418+Nat for *taz1Δ* QFA or G418+Hyg for *pot1-1* QFA). Liquid cultures were incubated at 30°C for two days without shaking and 384-format robotic spot tests were performed using a Biomek FX robot (Beckman *C*oulter (UK) Limited, High Wycombe, UK) equipped with a pintool magnetic mount and a 96-pin (2 mm diameter) pintool (V&P Scientific, Inc., San Diego, CA, USA). Photography, image analysis and modelling of fitness were performed as described previously [19]. Strip charts were generated using the stripchart function from the statistical programming software R [72].

### Manual Growth Assays

The strains were cultured to saturation in 2ml YE5S media with rolling at 30°C. A five or six-fold serial dilution using distilled water was then generated and spotted on YE5S plates. Strains were incubated at the indicated temperatures for 3–6 days before being photographed.

## Supporting Information

S1 Fig The 768 SGA colony format. Double mutants are arrayed in a 768 colony format with 308 deletion mutations arranged in pairs and surrounded by a neutral mutation (*mug134Δ*) in pairs (in blue). The green rectangular boxes are putative synthetic lethal interactions when pairs of double deletion mutations do not form colonies. Single yellow colonies are either pinning artefacts or an issue arising in an SGA.
(TIF)

S2 Fig *his3Δ* with *taz1Δ* small scale SGA using *HS* and *C* methods. Images of final selection plates from the *HS* and *C* methods are shown. The double mutants were photographed after growing them at 30°C for 3 days. Four plates (eight independent replicates, each row) were examined. The 16 diagonal black rectangular boxes indicate a proportion of double deletion pairs showing growth defects in all SGA plates. The red rectangular box highlights the *sde2Δ* location.
(TIF)

S3 Fig Quantitative comparison of *his3Δ* with *taz1Δ* small scale SGAs (A) Scatter plot for the *his3Δ* and *taz1Δ* small scale SGA using the *C* method. **(B)** Same as (A) but for the *HS* method.
(TIF)

S4 Fig Small scale *taz1Δ* QFA at 30°C QFA scatter plot comparing fitnesses (same as Fig 3B) of *his3Δ yfgΔ* and *taz1Δ yfgΔ* strains at 30°C (permissive temperature). (TIF)

S5 Fig Optimising temperature for *pot1-1* QFA Boxplots summarising quantitative fitness distributions for *pot1-1* query strains and the wild-type surrogate strains (*pot1* ^*+*^) at 30°C, 37°C, 38°C and 39°C (N = 44). (TIF)

S6 Fig Differences between *pot1-1* and *POT1* strain fitnesses observed after pinning are negligible. Boxplots summarising quantitative fitness distributions for *pot1-1* query strains and the wild-type surrogate strains (*pot1* ^*+*^) at 38°C, 39°C, 40°C and 41°C (N = 44).
(TIF)

S1 Supporting Information Description of the experiments carried out and the data listed within each of the types of raw data text file listed below. The raw data below can be used to replicate all of the plots and statistical analysis presented in the manuscript.
(PDF)

S2 Supporting Information Comparing *his3Δ* colony sizes, after haploid selection by cycloheximide, in large and small scale SGA screens. (TXT)

S3 Supporting Information Comparing *his3Δ* colony sizes, after haploid selection by heat shock, in large and small scale SGA screens. (TXT)

S4 Supporting Information Quantifying the strength of genetic interaction with *taz1Δ*, after haploid selection by heat shock, using small scale QFA screens. (TXT)

S5 Supporting Information Quantifying the strength of genetic interaction with *pot1-1*, after haploid selection by heat shock, using genome-wide QFA screens. (TXT)

S6 Supporting Information Measuring the size of *his3Δ* colonies genome-wide after haploid selection by cycloheximide. (TXT)

S7 Supporting Information Measuring the size of *his3Δ* colonies genome-wide after haploid selection by heat-shock. (TXT)

S8 Supporting Information Measuring the size of *his7Δ* colonies genome-wide after haploid selection by cycloheximide. (TXT)

S9 Supporting Information Measuring the size of *his7Δ* colonies genome-wide after haploid selection by heat-shock. (TXT)

S10 Supporting Information Measuring the size of *ura5Δ* colonies genome-wide after haploid selection by cycloheximide. (TXT)

S11 Supporting Information Measuring the size of *ura5Δ* colonies genome-wide after haploid selection by heat-shock. (TXT)

S12 Supporting Information Replicate fitness observations for QFA0068. (ZIP)

S13 Supporting Information Replicate fitness observations for QFA0069. (ZIP)

S14 Supporting Information Replicate fitness observations for QFA0088. (ZIP)

S15 Supporting Information Replicate fitness observations for QFA0089. (ZIP)

S16 Supporting Information Replicate fitness observations for QFA0015. (ZIP)

S17 Supporting Information Replicate fitness observations for QFA0018. (ZIP)

S18 Supporting Information Replicate fitness observations for QFA0065. (ZIP)

S19 Supporting Information Replicate fitness observations for QFA0067. (ZIP)

S1 Tables Strains and sample data. Excel spreadsheet containing the following tables as worksheets. Worksheet A: Strains used in the study. Worksheet B: List of gene deletions in the small scale library. Worksheet C: Raw data from the *taz1Δ* small scale QFA screen. Worksheet D: Raw data from the pot1-1 QFA screen.
(XLSX)
