# Supplementary figures and images for: Quantitative Fitness Analysis Identifies exo1∆ and Other Suppressors or Enhancers of Telomere Defects in Schizosaccharomyces pombe
Source: PLoS One. 2015 Jul 13;10(7):e0132240. doi: 10.1371/journal.pone.0132240 (PMC4500466; doi:10.1371/journal.pone.0132240)

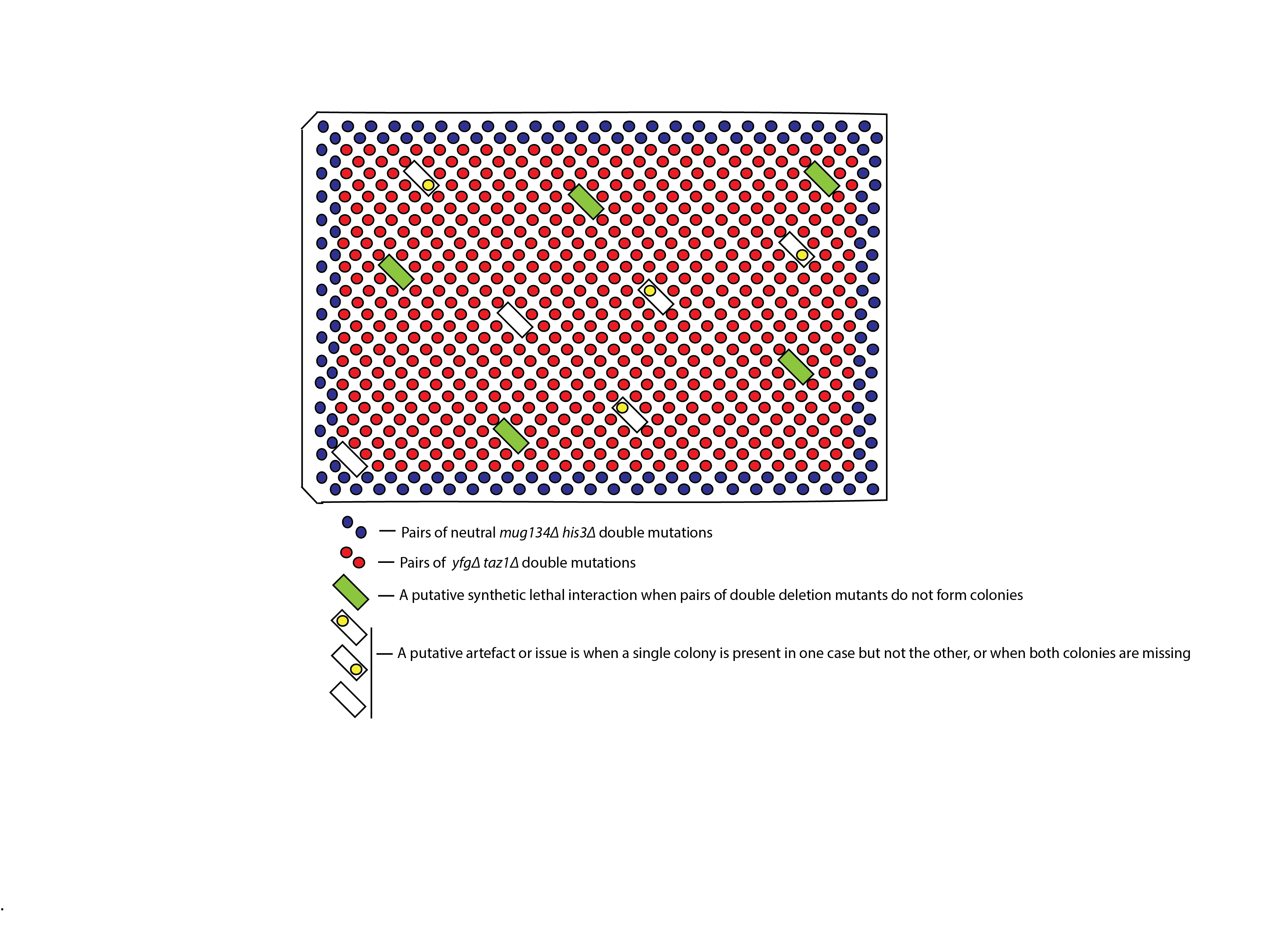

Supplement: S1 Fig — Double mutants are arrayed in a 768 colony format with 308 deletion mutations arranged in pairs and surrounded by a neutral mutation (mug134Δ) in pairs (in blue). The green rectangular boxes are putative synthetic lethal interactions when pairs of double deletion mutations do not form colonies. Single yellow colonies are either pinning artefacts or an issue arising in an SGA. (TIF) [file pone.0132240.s001.tif]

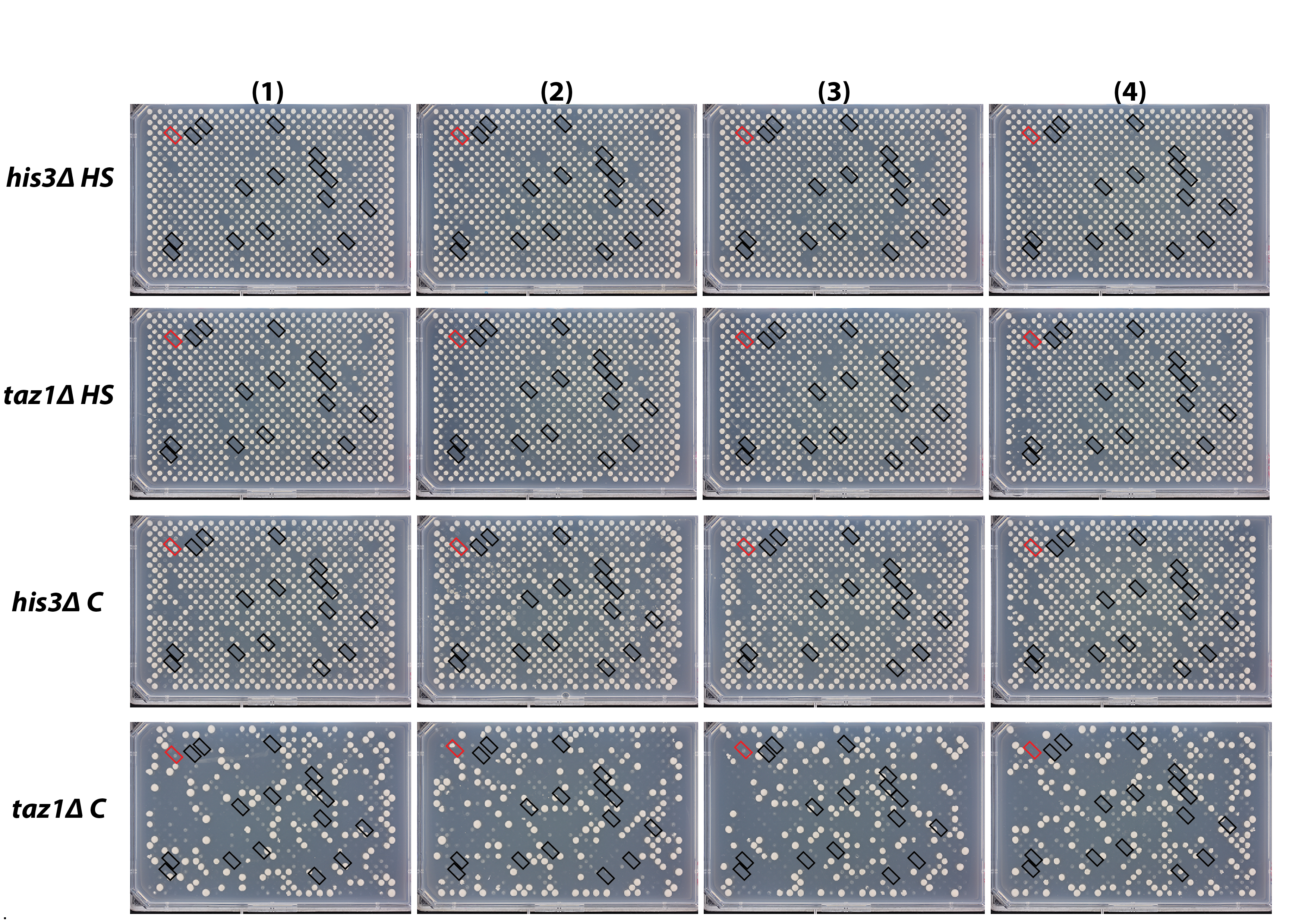

Supplement: S2 Fig — Images of final selection plates from the HS and C methods are shown. The double mutants were photographed after growing them at 30°C for 3 days. Four plates (eight independent replicates, each row) were examined. The 16 diagonal black rectangular boxes indicate a proportion of double deletion pairs showing growth defects in all SGA plates. The red rectangular box highlights the sde2Δ location. (TIF) [file pone.0132240.s002.tif]

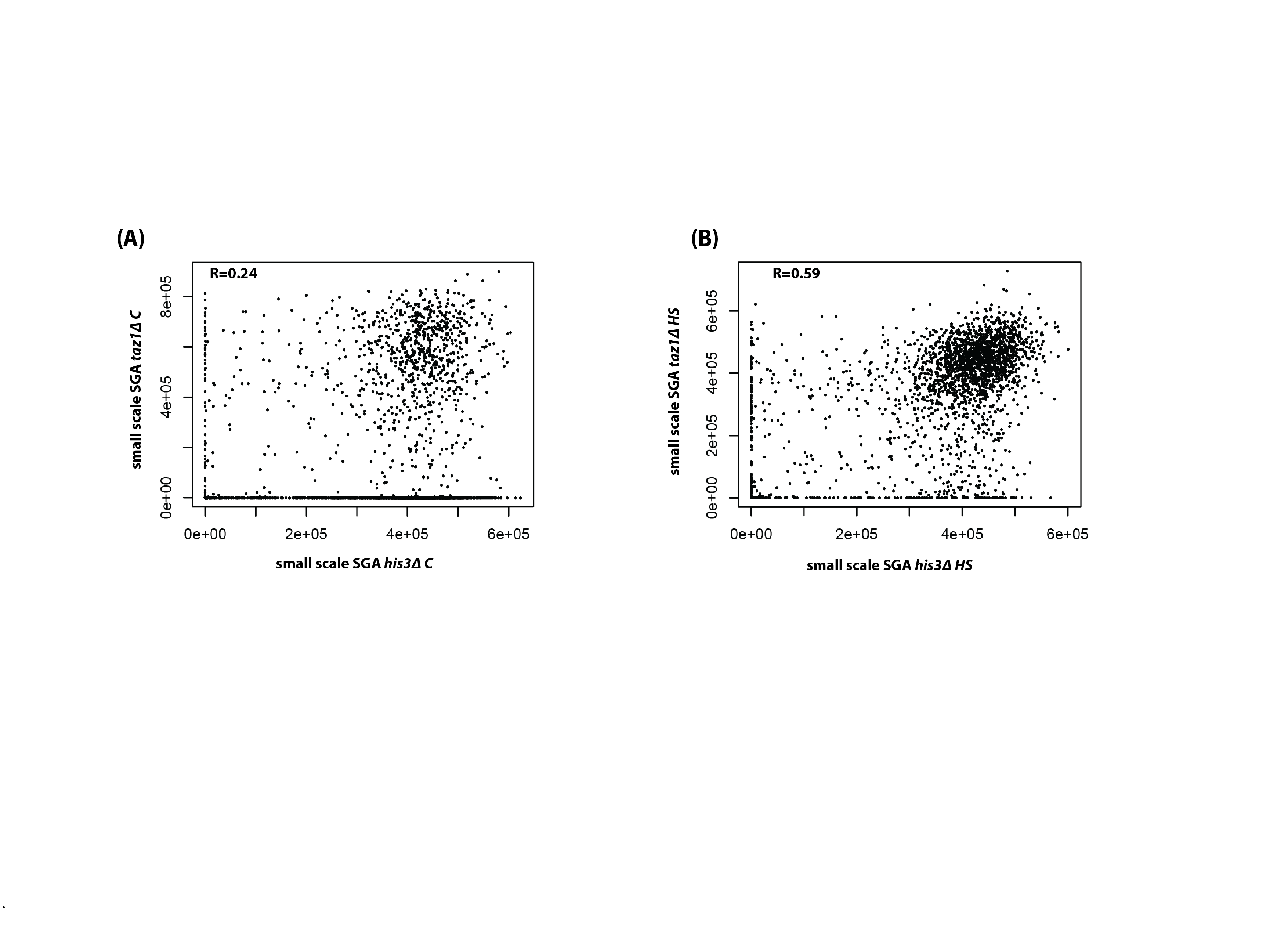

Supplement: S3 Fig — (B) Same as (A) but for the HS method. (TIF) [file pone.0132240.s003.tif]

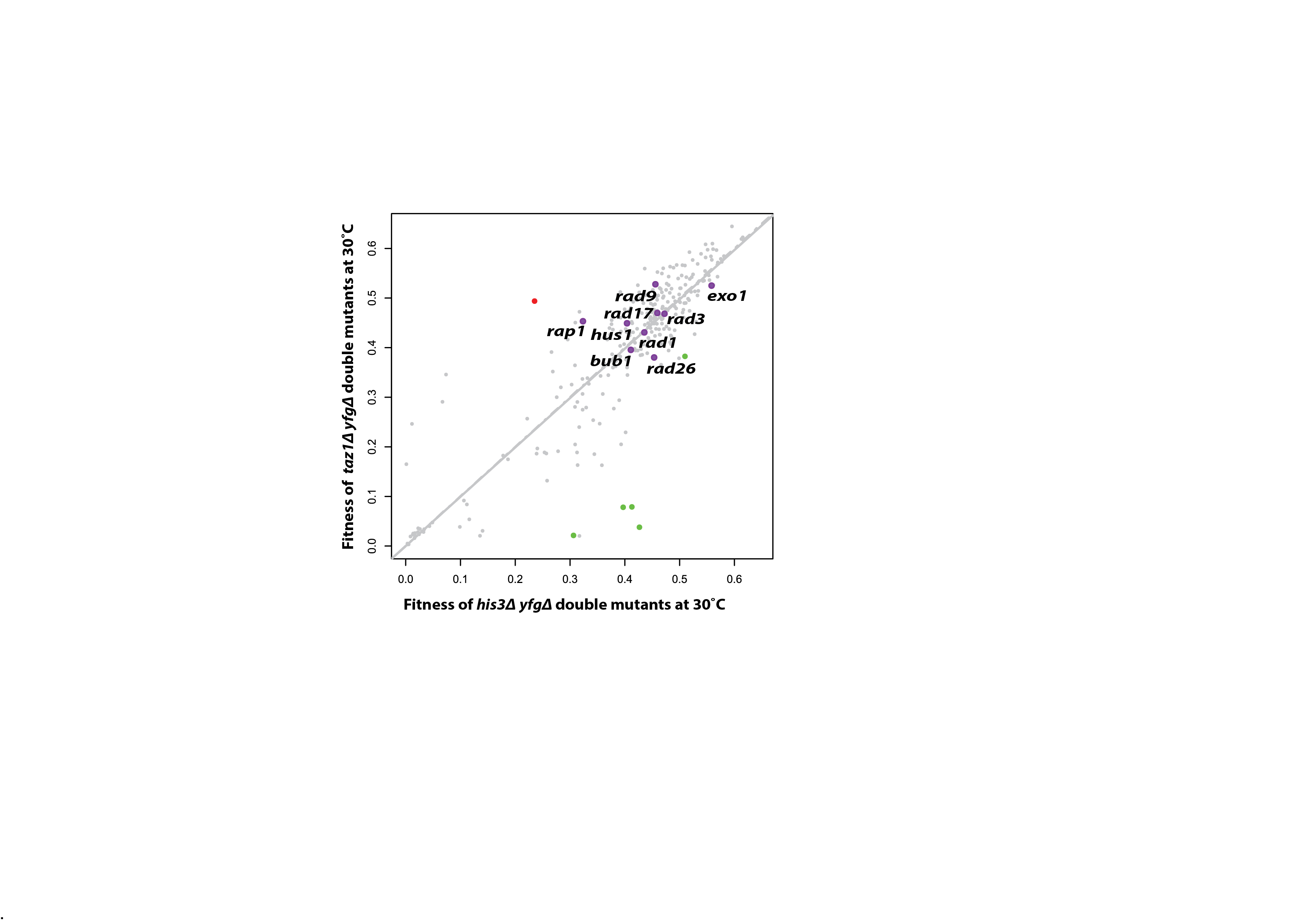

Supplement: S4 Fig — (TIF) [file pone.0132240.s004.tif]

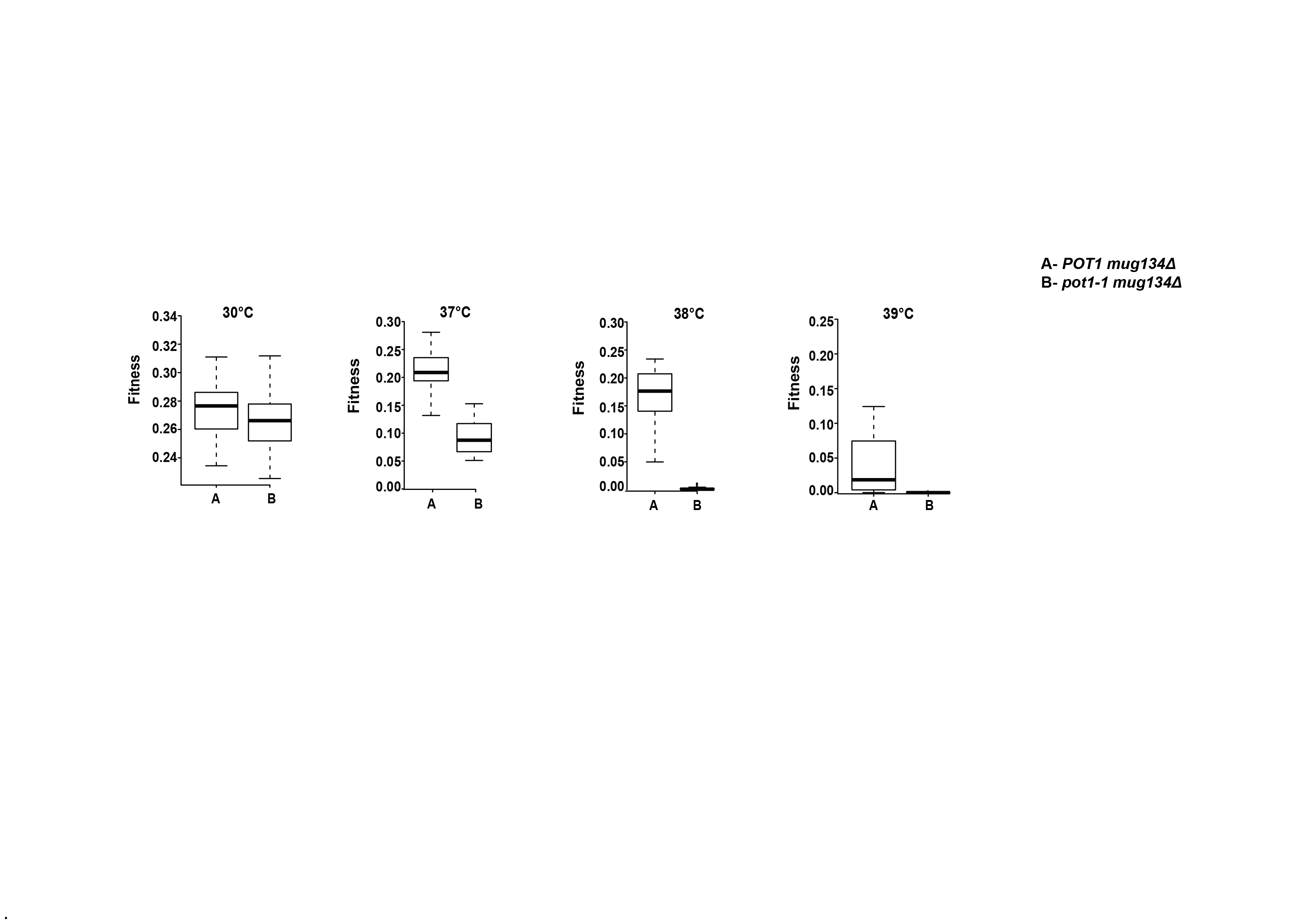

Supplement: S5 Fig — (TIF) [file pone.0132240.s005.tif]

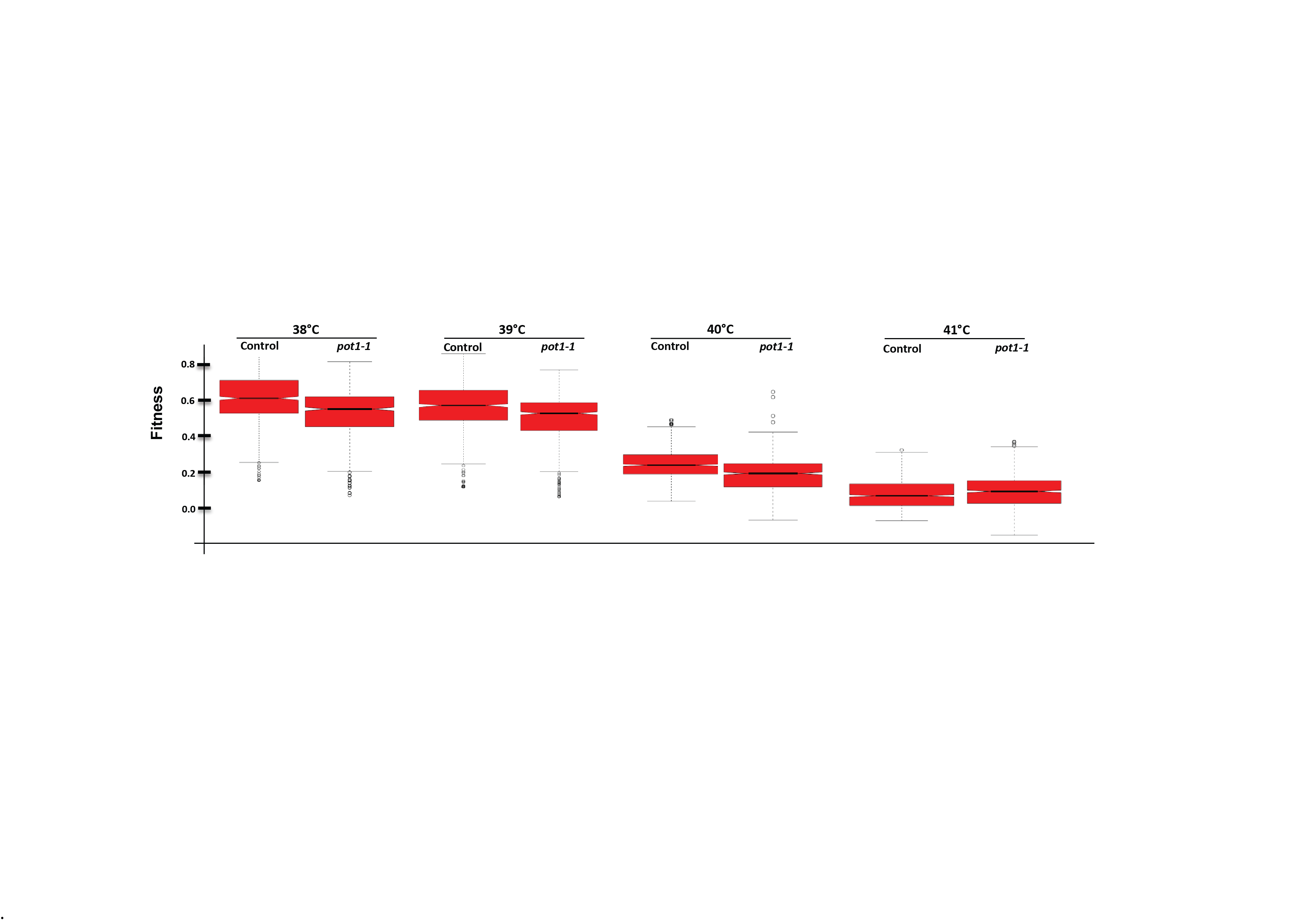

Supplement: S6 Fig — Boxplots summarising quantitative fitness distributions for pot1-1 query strains and the wild-type surrogate strains (pot1 +) at 38°C, 39°C, 40°C and 41°C (N = 44). (TIF) [file pone.0132240.s006.tif]
