# Supplementary material for: Quantitative Fitness Analysis Identifies exo1∆ and Other Suppressors or Enhancers of Telomere Defects in Schizosaccharomyces pombe
Source: PLoS One. 2015 Jul 13;10(7):e0132240. doi: 10.1371/journal.pone.0132240 (PMC4500466; doi:10.1371/journal.pone.0132240)
Supplement: S1 Supporting Information — The raw data below can be used to replicate all of the plots and statistical analysis presented in the manuscript. (PDF) [file pone.0132240.s007.pdf]

Supplementary Information

To allow plots and statistical analysis presented in this paper to be reproduced, we have included three types of file, tabulating fitnesses, fitness summaries and estimates of genetic interaction strength. The contents of each of these files are described briefly below. For more information, including how to generate each file type in your own experiment and how to convert from one file type to another, please see the documentation for the QFA R package (http://qfa.r-forge.r-project.org/).

Supplementary filenames include screen IDs (e.g. QFA0068) indicating specific experiments as follows:

Screen ID	Query strain	Temp. (°C)	Selection media	SGA method	Experiment scale	Pinning format
SGA0004	*his3Δ*	30	YE5S_CGN	*Cycloheximide*	Large scale	768
SGA0015	*his3Δ*	30	YE5S_CGN	*Cycloheximide*	Large scale	768
SGA0018	*his3Δ*	30	YE5S_GN	*Heat-shock*	Large scale	768
SGA0049	*his3Δ*	30	YE5S_GN	*Heat-shock*	Large scale	768
SGA0050	*his7Δ*	30	YE5S_CGN	*Cycloheximide*	Large scale	768
SGA0056	*ura5Δ*	30	YE5S_CGN	*Cycloheximide*	Large scale	768
SGA0058	*his7Δ*	30	YE5S_GN	*Heat-shock*	Large scale	768
SGA0059	*ura5Δ*	30	YE5S_GN	*Heat-shock*	Large scale	768
SGA0065	*his3Δ*	30	YE5S_CGN	*Cycloheximide*	Small scale	768
SGA0067	*his3Δ*	30	YE5S_GN	*Heat-shock*	Small scale	768
QFA0068	*his3Δ*	20	YE5S_GN	*Heat-shock*	Small scale	384
QFA0069	*taz1Δ*	20	YE5S_GN	*Heat-shock*	Small scale	384
QFA0088	*pot*$^+$	37	YE5S_GH	*Heat-shock*	Large scale	384
QFA0089	*pot1-1*	37	YE5S_GH	*Heat-shock*	Large scale	384

Media key: C: Cycloheximide, G: Geneticin (G418), N: Nourseothricin (clonNAT), H: Hygromycin, YE5S: Yeast extract 5 supplements

Individual Fitness (Fit.txt) files

Contain experimental metadata and a selection of different measures of fitness and parameter values for a population model (generalised logistic model) summarising growth curves for each spot on each plate of an experiment. Columns can be interpreted as follows:

Barcode - Unique plate identifier

Row - Row number (counting from top of image) of culture in rectangular gridded array

Col - Column number (counting from left of image) of culture in rectangular gridded array

ScreenID - Unique identifier for this QFA screen

Treatment - Conditions applied externally to plates (e.g. temperature(s) at which cultures were grown, UV irradiation applied, etc.)

Medium - Nutrients/drugs in plate agar

ORF - Systematic, unique identifier for genotype in this position in arrayed library

Screen.Name - Name of screen (identifies biological repeats, and experiment)

Library.Name - Name of library, specifying particular culture location

MasterPlate Number - Library plate identifier

Timeseries order - Sequential photograph number

Inoc.Time - User specified date and time of inoculation (specified in ExptDescription.txt file)

TileX - Culture tile width (pixels)

TileY - Culture tile height (pixels)

XOffset - x-coordinate of top left corner of rectangular tile bounding culture (number of pixels from left of image)

YOffset - y-coordinate of top left corner of rectangular tile bounding culture (number of pixels from top of image)

Threshold - Global pixel intensity threshold used for image segmentation (after lighting correction)

EdgeLength - Number of culture pixels classified as being microcolony edge pixels (useful for classifying contaminants in cultures grown from dilute inoculum)

EdgePixels - Number of pixels classified as culture on edge of square tile

RepQuad - Integer identifying which of the quadrants of a 1536 plate were used to inoculate the current 384 plate (set equal to 1 for all cultures for 1536 format for example)

K - Generalised logistic model carrying capacity

r - Generalised logistic model rate parameter

g - Generalised logistic model inoculum density (referred to in vignette as g_0)

v - Generalised logistic model shape parameter (set to 1 to recover logistic model)

objval - Objective function value at selected optimum

tshift - Shift applied to observation times before fitting logistic model (need to apply same shift before overlaying curve on expt. obs.). Default is zero (expt. starts at inoculation time specified in experimental description file), but if qfa.fit function is called with inocguess=NULL, then the start of experiment is redefined as the time of the first reliable density observation.

t0 - Time of first detectable cell density observation (i.e. above detectThresh)

d0 - Normalised cell density of first observation (be careful about condensation on plates when using this). Note this is not necessarily the density at t0.

nAUC - Numerical Area Under Curve. This is a model-free fitness estimate.

nSTP - Single Time Point fitness. Cell density at time STP, as estimated with approximating function. This is a model-free fitness estimate.

nr - Numerical estimate of intrinsic growth rate. Growth rate estimated by fitting smoothing function to log of data, calculating numerical slope estimate across range of data and selecting the maximum estimate (should occur during exponential phase).

nr_t - Time at which maximum slope of log observations occurs

maxslp - Numerical estimate of maximum slope of growth curve. Slope estimated by fitting smoothing function to untransformed data and calculating numerical slope estimate of smoothed version of data and selecting the maximum estimate (should occur approximately half way through growth). This fitness measure will be affected by both rate of growth and final colony size. Final colony size is expected to be strongly affected by competition between cultures.

maxslp_t - Time at which maximum slope of observations occurs

Client - Client for whom screen was carried out

ExptDate - A representative/approximate date for the experiment (note that genome-wide QFA screens typically take weeks to complete)

User - Person who actually carried out screen

PI - Principal investigator leading project that screen is part of

Condition - The most important defining characteristic of screen, as specified by user (e.g. the temperature screen was carried out at if screen is part of multi-temperature set of screens, or the query mutation if part of a set of screens comparing query mutations, or the drugs present in the medium if part of a set of drug screens)

Inoc - Qualitative identifier of inoculation type (e.g. "DIL" for dilute inoculum, "CONC" for concentrated). Used to distinguish between experiments carried out with different methods of inoculation.

Gene - Identifier for genotype at a particular location on an agar plate. Typically prefer unambiguous, systematic gene names here.

TrtMed - Combination of treatment and medium identifiers, specifying the environment in which the cells have grown

MDP - Maximum Doubling Potential: number of doublings in cell density observed from inoculum to carrying capacity, derived from logistic model parameters

MDR - Maximum Doubling Rate: intrinsic growth rate at t=0, derived from logistic model parameters MDRMDP - Product of MRD and MDP

glog_maxslp - maxslp derived from logistic model parameters

DT - Doubling time at t=0, derived from logistic model parameters

AUC - Area Under growh Curve up to AUCLim, derived from logistic model parameters

Fit - Copy of one of the fitness columns (typically MDRMDP) for further analysis

Genetic Interaction Strength (GIS.txt) files

Contain the evidence for genetic interaction strength estimates and estimates of the statistical significance of GIS. Also contain fitness summaries for both query and control experiment. Plotting QueryFitnessSummary (y-axis) against ControlFitnessSummary (x-axis) and fitting a linear regression, forced through the origin, to the resultant points, produces a fitness plot.

R package version - Version of software used for analysis

Summary type - Statistic used to summarise fitness replicates (e.g. mean or median)

Test type - Statistical test used to classify deviations of observed from predicted fitness as significant (e.g. t-test or Wilcoxon test)

x/y-axis treatment - Conditions experienced by plate (e.g. Temperature or UV irradiation)

x/y -axis medium - Contents of agar substrate x/y -axis screen ID: QFA0068

x/y -axis screen name - Experiment tag, typically more human-readable than Screen ID

x/y -axis libraries - Identifier for libraries used in this experiment (e.g. yeast knock-out collection)

x/y -axis client - Person who commissioned experiment

x/y -axis user - Person who carried out experiment

x/y -axis date - Approximate date experiment carried out

x/y -axis PI - Principal Investigator in charge of experiment

x/y -axis condition - Specification of the main factor (e.g. treatment, medium or genotype) for an experiment

x/y -axis inoculation type - Label indicating how cells were inoculated (e.g. CONC, DIL)

x/y -axis fitness definition - Which measure of fitness is summarised and used to calculate GIS

ORF - Unique strain genotype identifier (e.g. Y-number for yeast strains)

Gene - Human readable genotype identifier

P - p-value for significance of difference between control and query strain fitnesses

Q - q-value for significance of difference between control and query strain fitnesses. This is FDR corrected p-value

GIS - Genetic interaction strength. Deviation of (mean or median, depending on value of wctest) observed query strain fitness from expected fitness given control query strain fitness and a multiplicative model of genetic interaction.

QueryFitnessSummary - Summary statistic for all available replicate observations of query strain fitness (mean or median, depending on value of wctest).

ControlFitnessSummary - Summary statistic for all available replicate observations of control strain fitness (mean or median, depending on value of wctest).

QuerySE - Standard error on mean of query strain fitness observations

ControlSE - Standard error on mean of control strain fitness observations

TestType - Type of statistical test for significant difference carried out (i.e. Wilcoxon or t-test)

Type - Type of genetic interaction observed (suppressor, enhancer, positive, negative). This is assigned for strains with abs(GIS)>GISthresh and by comparing q-value with qthresh.

Fitness Report (FitnessReport) files

Similar to the GIS.txt files above, but only contain fitness summaries for an individual experiment. Individually, these files do not contain evidence for genetic interaction.

R package version - Version of software used for analysis

Treatment - Conditions experienced by plate (e.g. Temperature or UV irradiation)

Medium - Contents of agar substrate

Screen ID - Systematic experiment identifier (uniquely identifies one set of plates)

Screen name - Experiment tag, typically more human-readable than Screen ID

Libraries - Identifier for libraries used in this experiment (e.g. yeast knock-out collection)

Client - Person who commissioned experiment

User - Person who carried out experiment

PI - Principal Investigator in charge of experiment

Date - Approximate date experiment carried out

Fitness definition - Which measure of fitness is summarised

Condition - Specification of the main factor (e.g. treatment, medium or genotype) for an experiment

Gene - Standard gene name

ORF - Systematic gene name

MedianFit - Median fitness summary of all available replicates for gene

MeanFit - Mean fitness summary of all available replicates for gene

VarianceFit - Variance of fitness replicates for gene

NumRepeats - Number of replicate fitnesses available for gene

SEFit - Standard error of fitness replicates for gene
